# Editorial: Multi-Omics Approaches to Study Placental Development and Disease

**DOI:** 10.3389/fcell.2021.798966

**Published:** 2021-11-08

**Authors:** Geetu Tuteja, Michael J. Soares

**Affiliations:** ^1^ Department of Genetics, Development and Cell Biology, Iowa State University, Ames, IA, United States; ^2^ Institute for Reproduction and Perinatal Research, University of Kansas Medical Center, Kansas City, KS, United States; ^3^ Department of Pathology and Laboratory Medicine, University of Kansas Medical Center, Kansas City, KS, United States; ^4^ Center for Perinatal Research, Children’s Mercy Research Institute, Kansas City, MO, United States; ^5^ Department of Obstetrics and Gynecology, University of Kansas Medical Center, Kansas City, KS, United States

**Keywords:** trophoblast, placenta, multi-omics, pregnancy, syncytiotrophoblast, extravillous trophoblast, invasion, differentiation

The placenta carries out diverse roles that are critical for the establishment and maintenance of pregnancy, including anchoring the fetus to the uterine wall, transporting nutrients and oxygen to the fetus, eliminating waste, shielding the fetus from the maternal immune system, and producing hormones. Defects in placental development can lead to pregnancy complications that impact both the mother and fetus, yet our understanding of the mechanisms regulating placental cell differentiation and other placental processes, especially at the systems level, remains limited. The research topic on “Multi-Omics Approaches to Study Placental Development and Disease” in *Frontiers in Cell and Developmental Biology* includes a series of 10 articles. Each article describes at least one omics-based approach used to aid in our understanding of placental development, and many articles provide novel candidates that could be further characterized for a functional role in the placenta.

For those who are not familiar with how omics-approaches have been used to study placental development, this issue includes three comprehensive review articles. One review article by Lee and Kim describes *cis-*regulation and *trans-*regulation studies in mouse and human placenta, and also describes recent *in vitro* human trophoblast model systems. The second review article by Jaremek et al. focuses on syncytiotrophoblast (STB), which facilitate human embryo implantation and later in development are the placenta cells that are in direct contact with maternal blood and facilitate nutrient transport. In addition to describing STB development, Jaremek et al. describe diverse omics-approaches that have been used to study STB, including metabolomics and proteomics. Rather than focusing on normal mechanisms of development, the third review article by Rosenfeld describes how endocrine disrupting chemicals and other environmental toxicants impact placental development by disrupting placental gene expression, DNA methylation patterns, and metabolomic profiles.

The placenta is composed of multiple types of trophoblast cells, and even the same type of trophoblast can have different properties during development. It is therefore important to identify and distinguish trophoblast cells that exhibit unique properties. To this end, this issue includes a research article by Morey et al. that profiles extravillous trophoblast from 1st trimester and term placenta to identify gene networks and novel candidates that could regulate early extravillous trophoblast differentiation or extravillous trophoblast maturation. In another article by Khan et al., single-nucleus RNA-seq was used on a trophoblast cell culture model, and two distinct populations of syncytiotrophoblast were identified, requiring further investigation as to how these populations relate to early human placentation.

In addition to understanding changes in expression of protein-coding genes during placental development or in pregnancy complications, it is also necessary to understand changes in the expression of non-coding RNAs, which are known to have regulatory functions and are less well studied in the placenta. This issue includes two studies that focus on identification of non-coding RNAs that could be important for placenta function. In the first article by Inno et al., microRNA expression was assessed in normal human placentas from all three trimesters, as well as placentas obtained from women with different pregnancy disorders. In addition to relating the microRNA data to mRNA expression, the study by Inno et al. highlights several specific microRNAs that may regulate placental development. In the second article by Chu et al., non-coding RNAs and mRNAs that are misregulated when primary human trophoblast differentiation was hindered were identified, and a co-expression network constructed to understand the relationship between different RNAs in the trophoblast differentiation system. It is of note that another article in this issue, by Rosario et al. could add yet another layer to our understanding of primary human trophoblast differentiation, as the secretome was assayed in these cells. Additional studies aimed at understanding the profiles of secreted proteins in the placenta are necessary, as transcriptome data alone will not give a full understanding of the complex regulatory processes taking place in the placenta.

While the previous studies use omics-approaches to identify novel candidates associated with placental development, omics-approaches can also be used to better understand the role of specific genes in the placenta. For example, Georgiadou et al. found that when PCBP2, an RNA splicing complex protein, was knocked-down in HTR-8/SVneo cells, global mRNA expression levels were not impacted, although, interestingly, the splicing pattern of genes involved in cellular organization, maintenance, and proliferation were impacted. In another article published by Rosario et al. in this issue, mTORC2, which regulates amino acid and folate transport in the placenta, was silenced in primary human trophoblast. Analysis of genes differentially regulated upon mTORC2 silencing led to the identification of a link between mTOR signaling, angiogenesis, micronutrient transport, and inflammation.

In summary, the application of omics-based technologies in the human placenta as well as trophoblast model systems have, and will continue, to provide a more complete picture of genes and pathways important for placental development and disease.

